# Cardiovascular risk factors among patients with chronic kidney disease attending a tertiary hospital in Uganda

**DOI:** 10.5830/CVJA-2015-045

**Published:** 2015

**Authors:** Christopher Babua, Robert Kalyesubula, Emmy Okello, Barbara Kakande, Elias Sebatta, Michael Mungoma, Charles Kiiza Mondo

**Affiliations:** Department of Medicine, Gulu University, Gulu, Uganda; Department of Medicine, College of Health Sciences, Makerere University, Kampala, Uganda; Department of Medicine, College of Health Sciences, Makerere University, Kampala, Uganda; Department of Medicine, College of Health Sciences, Makerere University, Kampala, Uganda; Department of Medicine, College of Health Sciences, Makerere University, Kampala, Uganda; Department of Medicine, College of Health Sciences, Makerere University, Kampala, Uganda; Department of Medicine, College of Health Sciences, Makerere University, Kampala, Uganda

**Keywords:** CVD risk factors, chronic kidney disease

## Abstract

**Introduction:**

Chronic kidney disease (CKD) is a risk factor for the development of cardiovascular disease, which is the primary cause of morbidity and mortality in patients with CKD. Local data about cardiovascular risk factors among CKD patients is generally scanty.

**Objective:**

To determine the prevalence of the common cardiovascular risk factors among patients with CKD attending the nephrology out-patient clinic in Mulago national referral hospital in Uganda.

**Methods:**

This was a cross-sectional study in which 217 patients with a mean age of 43 years were recruited over a period of nine months. Data on demographic characteristics, risk factors for cardiovascular disease, complete blood count, renal function tests/electrolytes, and lipid profiles were collected using a standardised questionnaire.

**Results:**

One hundred and eleven (51.2%) of the participants were male. Hypertension was reported in 90% of participants while cigarette smoking was present in 11.5%. Twenty-two participants (10.2%) were obese and 16.1% were diabetic. A total of 71.9% had a haemoglobin concentration < 11 g/dl, with the prevalence of anaemia increasing with advancing renal failure (*p* < 0.001); 44.7% were hypocalcaemic and 39.2% had hyperphosphataemia. The prevalence of abnormal calcium and phosphate levels was found to increase with declining renal function (*p* = 0.004 for calcium and *p* < 0.001 for phosphate).

**Conclusion:**

This study demonstrated that both traditional and non-traditional cardiovascular risk factors occurred frequently in patients with CKD attending the nephrology out-patient clinic at Mulago Hospital.

## Introduction

Cardiovascular disease is the primary cause of morbidity and premature mortality in chronic kidney disease patients.^1^,^2^ The high risk of cardiovascular morbidity and mortality in end-stage renal disease (ESRD) is a well-established fact.^3^ However a high rate of both fatal and non-fatal cardiovascular events has been observed in patients with earlier stages of chronic kidney disease (CKD).^4^ Most of the current guidelines now regard CKD as a cardiovascular risk equivalent.^5^

Traditional cardiovascular risk factors, those risk factors that predict ischaemic heart disease outcomes in the general population, have been reported to occur commonly in patients with CKD.^5^-^7^ These include hypertension, cigarette smoking, diabetes, dyslipidaemia and older age. The number of cardiovascular risk factors appears to correlate with the severity of kidney dysfunction.^7^

Non-traditional cardiovascular risk factors are CKD related and increase in frequency as renal function declines. They are thought to contribute to the cardiovascular risk excess in CKD patients compared with the general population.^3^ These factors include anaemia, abnormal calcium/phosphorus metabolism, malnutrition, hypo-albuminaemia, hyperhomocysteinemia, inflammation, oxidant stress, insulin resistance, altered renin– angiotensin axis and endothelial dysfunction.^8^

Despite the fact that cardiovascular diseases are a major cause of morbidity and mortality in patients with CKD, data on cardiovascular risk factors among CKD patients are generally lacking from low-resource countries such as Uganda. We conducted a study to determine the prevalence of the known cardiovascular risk factors among patients with CKD attending Mulago Hospital, a tertiary healthcare facility and university teaching hospital.

## Methods

We conducted a cross-sectional study between June 2012 and February 2013 at Mulago Hospital in Kampala, Uganda. The hospital also doubles as the teaching hospital for Makerere University’s College of Health Sciences and serves the 33 million people of Uganda as well as referrals from the neighbouring Eastern Democratic Republic of Congo and the Republic of Southern Sudan.

We consecutively recruited a total of 217 adults with CKD, aged 18 years and older. CKD was defined as kidney damage for three or more months, as confirmed by kidney biopsy or proteinuria, with or without a decrease in glomerular filtration rate (GFR); or GFR < 60 ml/min/1.73 m^2^ for three or more months, with or without kidney damage.^1^ Patients who had had any form of renal replacement therapy (haemodialysis, peritoneal dialysis or renal transplant) were excluded from the study. Ethical approval was obtained from the School of Medicine Research and Ethics Committee of the College of Health Sciences, Makerere University.

A standardised, pre-tested questionnaire was used to collect data on sociodemographic characteristics, medical history, laboratory test parameters, electrocardiography (ECG) and echocardiography variables, and physical signs, with an emphasis on cardiovascular risk factors. Six traditional cardiovascular risk factors, including male gender, older age, cigarette smoking, obesity, diabetes mellitus, hypertension (defined according to JNC 7),^9^ and dyslipidaemia [elevated non-high-density lipoprotein cholesterol > 130 mg/dl (3.37 mmol/l)], and two non-traditional risk factors, anaemia and abnormal calcium/ phosphate metabolism were the focus of this study.

Laboratory tests focused on levels of creatinine, urea, albumin, total cholesterol, triglycerides, high-density lipoprotein (HDL) cholesterol, low-density lipoprotein (LDL) cholesterol, phosphorus, calcium, haemoglobin and proteinuria. Haemoglobin concentration was determined using Celltac E, an automated CBC machine. Clinical chemistry tests were performed using Cobas 6000, an automated analyser from Roche pharmaceuticals.

Resting ECGs were carried out using the Schillar ECG Recorder, (Basal, Switzerland). Echocardiograms were done using the Vivid 7 Dimension, GE Medical Systems (Horten, Norway) according to American Society of Echocardiography guidelines. GFR was estimated for all study participants using the Cockcroft–Gault equation. Non-HDL cholesterol and body mass index were calculated using standard methods.

## Statistical analyses

Data were double entered into epidata version 3.1 and exported to STATA version 10 (after validation) for analysis. Results were expressed as percentages and means with standard deviations, and presented in tables and graphs. Chi-squared tests were used to determine associations (declining renal function versus riskfactor profiles). Results were statistically significant when the *p*-value was < 0.05.

## Results

**Figure 1.**
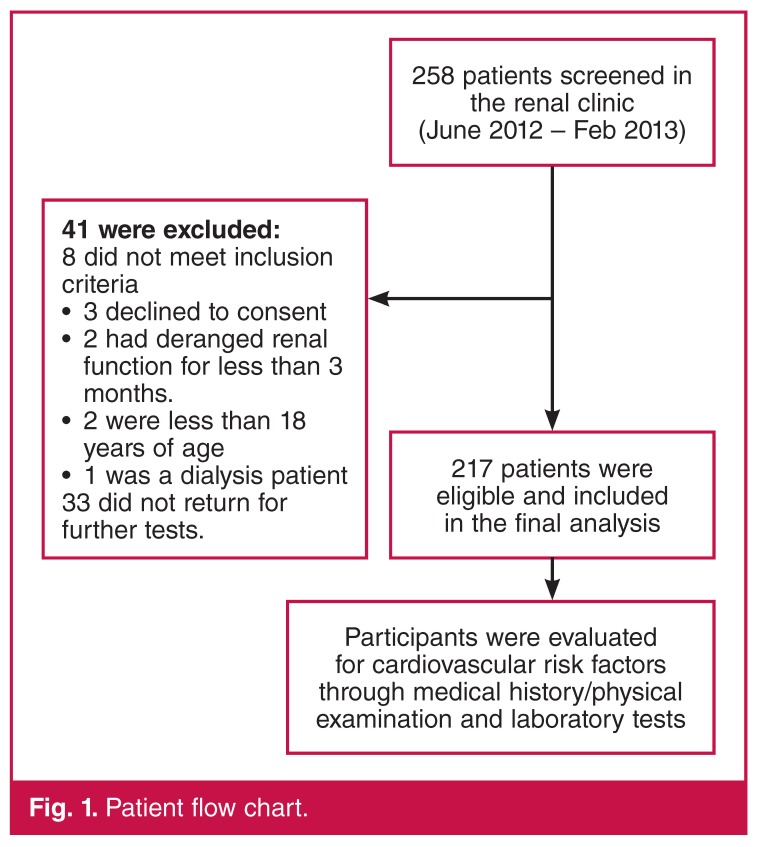
Patient flow chart.

A total of 258 patients were screened over a period of nine months. Forty-one were excluded from the study for various reasons (Fig. 1). One hundred and eleven (51.2%) of the participants were male. The mean age of study participants was 42.8 years (95% CI = 40.6–44.9).

**Figure 2.**
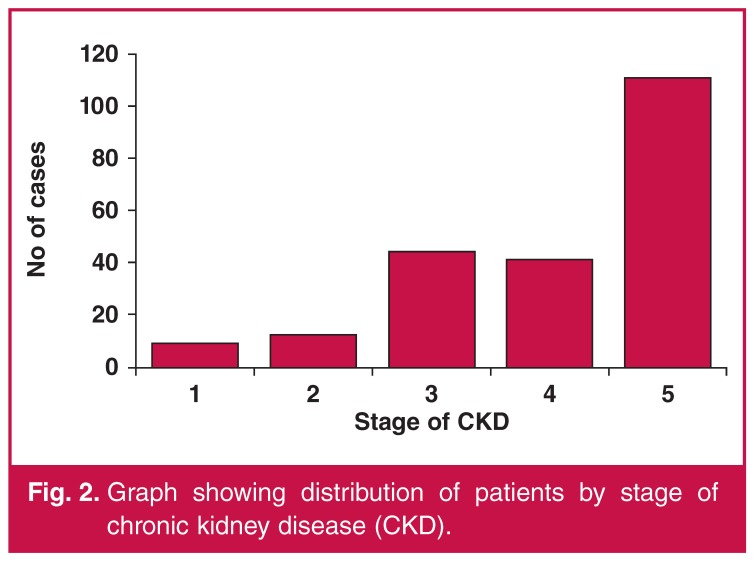
Graph showing distribution of patients by stage of chronic kidney disease (CKD).

About half of the patients had ESRD (111, 51.2%) (Fig. 2). A total of 184 patients (84.8%) had proteinuria. One hundred and sixty-two subjects (74.65%) had had a non-reactive HIV antibody test within the three months prior to recruitment, 32 patients (14.75%) were HIV positive, and the remaining 23 (10.60%) had had no evidence for taking the HIV test in the previous three months. The patient characteristics are summarised in Table 1.

**Table 1:** Demographic and clinical characteristics of study participants

*Characteristic*	*Frequency (n = 217)*	*Percentage (%)*
Age		
< 45 years	124	57.14
Gender		
Female	106	48.85
Stage of CKD		
1 (GFR ≥ 90 ml/min/m^2^)	9	4.15
2 (GFR 60–89 ml/min/m^2^)	12	5.53
3 (GFR 30–59 ml/min/m^2^)	44	20.28
4 (GFR 15–29 ml/min/m^2^)	41	18.89
5 (GFR < 15 ml/min/m^2^)	111	51.15
Proteinuria		
present	184	84.79
HIV antibody test status		
Non-reactive	162	74.65
Reactive	32	14.75
Not available	23	10.60

GFR: glomerular filtration rate, HIV: human immunodeficiency virus.

Twenty-five patients (11.5%) were either current smokers or had a history of tobacco smoking. There was a higher prevalence of cigarette smoking among males (18.9 vs 3.8) and this was statistically significant (*p* < 0.001). The prevalence of hypertension was 90%, with 88% of patients on treatment but only 24% had their blood pressure under control (Table 2). Diabetes prevalence was 16.1% and 22 patients (10.1%) were obese (BMI ≥ 30 kg/m^2^). Despite the fact that 89 patients (41%) had elevated non-HDL cholesterol of ≥ 130 mg/dl (3.37 mmol/l), only nine patients (4.2%) were on statin lipid-lowering therapy.

**Table 2:** Traditional and non-traditional cardiovascular risk factors among CKD patients attending Mulago renal clinic

*Variable*	*Frequency (n = 217)*	*Percentage (%)*
Cigarette smoking	25	11.5
Hypertension	196	90.0
Hypertension on treatment	191	88.0
Hypertension under control on treatment	46	24.0
Diabetes mellitus	35	16.2
Body mass index (kg/m^2^)		
< 18.5	20	9.2
18.5–24.9	113	52.1
25–29.9	62	28.6
≥ 30	22	10.1
Non-HDL cholesterol (mg/dl) (3.37 mmol/l)		
≥ 130	89	41.0
Haemoglobin concentration (g/dl)		
< 11	156	71.9
11–12	30	13.8
> 12	31	14.3
Serum calcium < 2.2 mmol/l	97	44.7
Serum phosphate > 1.5 mmol/l	85	39.2

HDL: high-density lipoprotein.

One hundred and fifty-six patients (71.9%) had haemoglobin concentrations < 11 g/dl. Only three patients (1.4%) were on weekly anaemia treatment with erythropoietin and iron sucrose, as recommended by the United States NKF-KDOQI. A large proportion, 156 patients (71.9%) were on oral iron and folate therapy.

Ninety-seven patients (44.70%) were found to have hypocalcaemia (calcium < 2.2 mmol/l) and 85 (39.17%) had serum phosphate concentrations above the reference range (0.9– 1.5 mmol/l). The cardiovascular risk factors are summarised in Table 2.

**Figure 3.**
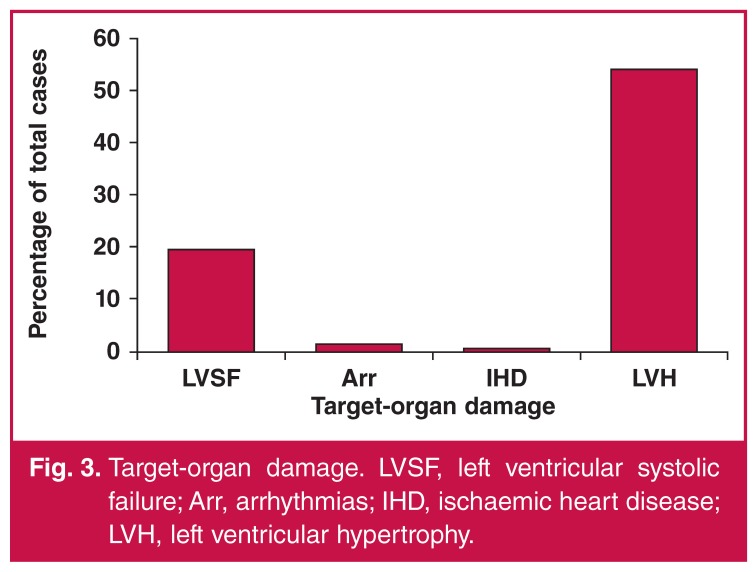
Target-organ damage. LVSF, left ventricular systolic failure; Arr, arrhythmias; IHD, ischaemic heart disease; LVH, left ventricular hypertrophy.

All study participants underwent resting ECG and two-dimensional echocardiography. Echocardiographically determined left ventricular hypertrophy (interventricular septum and/or left ventricular posterior wall thickness > 11 mm in diameter) was present in 54% of the participants, followed by left ventricular systolic failure (ejection fraction < 45%) in 19.4%. Ischaemic heart disease and malignant cardiac arrhythmias were less common (Fig. 3).

## Discussion

The major findings of this study were that (1) there was a high prevalence of both traditional and non-traditional risk factors for cardiovascular disease in our patients with CKD, (2) the majority of patients in this study were in advanced renal failure (stage 3–5), including more than half in ESRD (111, 51.2%); this represents two possibilities, including late presentation as well as limited access to renal replacement therapy, and (3) the severity and/or frequency of these factors increases with advancing stage of CKD (declining renal function) (Table 3).

**Table 3:** Variation of cardiovascular risk factors across the different CKD stages

	*CKD stage*					
	1	2	3	4	5	
*Variable*	*(n = 9)*	*(n = 12)*	*(n = 44)*	*(n = 41)*	*(n = 111)*	*p-value*
Hypertension, n (%)	4 (44.4)	8 (66.7)	41 (93.2)	39 (95.1)	99 (89.2)	< 0.001
Obesity, n (%)	0 (0.00)	2 (16.7)	3 (6.8)	6 (14.6)	11 (9.9)	0.797
Non-HDL-C > 130 mg/dl (3.37 mmol/l), n (%)	6 (66.7)	5 (41.7)	21 (47.7)	19 (46.3)	38 (34.2)	0.412
Diabetes, n (%)	1 (11.11)	1 (8.33)	8 (18.18)	8 (19.81)	17 (15.32)	0.871
Haemoglobin < 11 g/dl, n (%)	2 (22.2)	4 (33.3)	27 (61.4)	29 (70.7)	94 (84.7)	< 0.001
Calcium < 2.2 mmol/l, n (%)	0 (0.00)	4 (33.3)	11 (25)	17 (41.5)	65 (58.6)	0.004
Phosphate > 1.5 mmol/l, n (%)	1 (11.1)	1 (11.1)	8 (18.2)	11 (26.8)	64 (57.7)	< 0.001

CKD: chronic kidney disease, HDL-C: high-density lipoprotein cholesterol.

The prevalence of hypertension in this study was similar to the high prevalence of hypertension in other studies on patients with CKD. These include a study of 100 patients with CKD at the University of Nigeria teaching hospital, in which the prevalence of hypertension was found to be 85.2% at the first nephrology consultation.^10^ A similarly high prevalence of hypertension, at 72.6%, was found in Albanian patients with CKD.^11^ These findings show that there is a high burden of hypertension in CKD patients, regardless of the different patient populations studied. These figures are however four times those of the general population, where hypertension prevalence was at 20%.^12^,^13^

The available studies from sub-Saharan Africa however cannot determine with certainty whether hypertension is a cause or effect of CKD due to various limitations, such as study design, lack of histological data for participants, as well as late presentation of patients. In our study, the majority were in stage 4 and 5. This finding however underscores the importance of appropriate management of high blood pressure in patients with CKD.

The prevalence of diabetes in this study was similar to the 14.8% prevalence found among CKD patients in a Nigerian study.^10^ The similar prevalence is probably due to similarity of study settings as well as similarities in characteristics of study participants (e.g. age, race). However the prevalence of diabetes in this study was about four times the national prevalence of 4% in 2006,^14^ and 2.9% in 2011.^13^ This higher prevalence of diabetes among patients with CKD compared with the general population may reflect the significance of diabetes as an aetiological factor for CKD in Uganda.

The prevalence of smoking in this CKD population was similar to that of the general population, according to World Bank figures.^15^ Although data from Western countries suggest that traditional cardiovascular risk factors, including cigarette smoking, are highly prevalent in CKD populations,^5^-^7^ data for CKD patients in similar settings are scarce. As the incidence of a myocardial infarction is increased six-fold in women and three-fold in men who smoke at least 20 cigarettes per day compared to subjects who never smoked,^16^ smoking cessation in patients with CKD should be part of the standard management.

The burden of anaemia demonstrated in this study was similar to the high burden found in CKD populations elsewhere, including one done by Gjata *et al*. in Albania that showed a 100% frequency,^11^ and another from Nigeria where a high proportion of CKD patients with left ventricular hypertrophy (LVH) were found to be anaemic.^10^ The high prevalence of anaemia in all these settings is probably due to the similarity of pathogenesis, lack of erythropoietin, the most important factor in anaemia of CKD. Erythropoietin production/secretion declines with advancing renal failure in all cases of CKD, regardless of cause, hence the similarity in prevalence across the different settings. Anaemia in CKD has been associated with poorer cardiovascular outcomes, including heart failure, LVH and increased rates of morbidity and mortality.^17^-^19^

We observed the prevalence of HIV/AIDS of 14.75% to be twice that in the general population (7.2%),^20^ and HIV is a risk factor for CKD. This makes our study population different from those in Western countries with less incidence of HIV, suggesting that HIV/AIDS could be an emerging ‘non-traditional’ risk factor for CVD.

There were limitations to our study. Our inability to do renal biopsy meant that analysis for CKD was done as a block as opposed to clusters according to aetiology. The Cockroft– Gault formula was used for estimation of GFR instead of the more accurate MDRD or CKD-EPI methods. This is because most of the drug dosing is still based on the Cockcroft–Gault formula, and most doctors in resource-limited settings do not have access to the internet-based calculation of MDRD and CKD-EPI, which would have made applicability of our study findings less desirable. Some biomarkers of poor cardiovascular outcomes, regarded as non-traditional cardiovascular risk factors (inflammation, oxidative stress, sympathetic activation, hyperhomocysteinaemia, and endogenous digitalis-like factors) were not measured due to resource limitations. The criteria for evaluating ischaemic heart disease were weak.

## Conclusion

This study demonstrated the common occurrence of cardiovascular risk factors among CKD patients attending a Ugandan national referral hospital. It also showed that the prevalence of some of the risk factors (hypertension, anaemia, hypocalcaemia and hyperphosphataemia) increased with advancing stage of CKD. Furthermore it indicated late presentation of patients in advanced renal failure.
